# Diagnostic Test Accuracy of First-Void Urine Human Papillomaviruses for Presence Cervical HPV in Women: Systematic Review and Meta-Analysis

**DOI:** 10.3390/ijerph182413314

**Published:** 2021-12-17

**Authors:** Peter Bober, Peter Firment, Ján Sabo

**Affiliations:** 1 Department of Medical and Clinical Biophysics, Faculty of Medicine, University of P.J. Šafárik in Košice, Trieda SNP 1, 04011 Košice, Slovakia; jan.sabo@upjs.sk; 2 Department of Anaesthesiology and Intensive Medicine, FNsP J. A. Reimana Prešov, Jána Hollého 5898/14, 08181 Prešov, Slovakia; firment@fnsppresov.sk

**Keywords:** human papillomavirus, HPV DNA, cervical cancer, CIN, first-void urine

## Abstract

First-void urine usually contains exfoliated cells of the debris and mucus from the female genital organs and cervix, i.e., high concentration of human papillomavirus deoxyribonucleic acid (HPV DNA). We conducted a meta-analysis of published data and determined an accuracy of HPV detection in first-void urine compared to the women’s cervix. According to Preferred Reporting Items for Systematic Reviews and Meta-Analyses (PRISMA) guidelines, we carried out a comprehensive literature search. Eligible articles published from 2011 until 2021 were gathered by searching Embase, PubMed and Cochrane Library Central databases. The patient selection, index test, standard test, and patient flow were the factors involved in quality evaluation. A meta-analysis of 15 studies (3412 women) based on 5054 potential records was conducted. Pooled sensitivity for high-risk HPV detection in urine of 78% (70–84%) and specificity of 89% (81–94%) were calculated. Any HPV detection in urine of 87% (74–94%) and 91% (83–96%) were pooled sensitivity and specificity, respectively. HPV 16 and 18 had a pooled sensitivity of 77% (76–77%) and specificity of 98% (98–98%). Meta-analysis indicated variations between the pooled specificities and sensitivities. In meta-regression analysis, a heterogeneity in accuracy by using covariates (bias in patient selection, purpose, sample timing, storage temperature and HPV detection method) were not detected. Our meta-analysis demonstrates the accuracy of detection of HPV in urine for the presence of cervical HPV. Although progress is continuously made in urinary HPV detection, further studies are needed to evaluate and to improve the accuracy of the first-void urine test in order to be comparable with other screening methods.

## 1. Introduction

Is widely known that HPV is the primary cause of cervical cancer [1]. Cervical cancer presents the fourth-most cause of cancer deaths in women worldwide [2]. HPV is detected in almost all cervical cancer biopsies with more than 90% presence in high-grade squamous intraepithelial lesions (HSIL) [3]. More than 200 genotypes of HPV have been identified to date [4]. Of them, HPV16 and HPV18 represent the high-risk oncogenic genotypes, as they cause approximately 70% of nearly all cervical cancer [5,6,7].

A major impediment to controlling cervical cancer is lack of attendance for screening, i.e., in those countries without well-developed screening programs, from 50% to more than 80% of women are not screened [8]. In addition, in countries with well-organised screening programmes, half of all potentially detectable carcinomas are found in women who have not attended screening programmes [9].

There has been a drastic decline in the incidence, as well as the mortality, of cervical cancer worldwide since the introduction of the Pap test [10,11]. However, screening strategies for cervical cytology or Papanicolaou (Pap) tests requires uncomfortable and invasive pelvic examinations. Moreover, healthcare providers find it time-consuming and it cannot be carried out easily in resource-poor settings [12,13]. Additionally, cervical cytology can be susceptible as a result of technical or subjective errors, due to low sensitivity and false negative results [14,15].

There has been a great deal of interest lately in using urine as a liquid biopsy for HPV DNA testing, and this has increased due to observation of high correlations between urine and cervical HPV infections [16,17,18,19,20]. Urine samples are a good option for self-sampling screening since they are cheap, noninvasive and simple to collect [21,22]. The HPV test using urine appears to be an effective method for detecting HPV infection, so there is a possibility that it could be used for women who do not participate in routine screenings [23]. 

Urine voiding in the first part (first-void urine) usually contains exfoliated cells of the debris and mucus from the female genital organs and cervix, i.e., the first-void urine contains higher concentrations of HPV DNA than midstream urine. According to this theory, the identification of biomarkers in first-void urine, as well as HPV DNA, can be used to screen for (pre)cervical cancer [24].

Therefore, we conducted a systematic review and meta-analysis to determine the accuracy of detection of HPV in first-void urine compared with the cervix in women.

## 2. Materials and Methods

According to recommended methods, a meta-analysis and systematic review was conducted in compliance with Preferred Reporting Items for Systematic Reviews and Meta-Analyses (PRISMA) guidelines [25].

### 2.1. Criteria for Search and Eligibility

A literature review for the past 10 years (from January 2011 up to May 2021) in the three databases: Embase and Cochrane library (Title/Keywords/Abstracts) and PubMed (Title/Abstracts) was conducted. In each database, using Boolean logic, we searched for the following terms: (HPV or hrHPV or human papillomavir *) OR (HPV or hrHPV or human papillomavir *) AND (test * or assay * or genotyping or typing or detection or amplification) OR (HPV or hrHPV or human papillomavir *) AND (deoxyribonucleic or ribonucleic or nucleic or DNA or RNA or mRNA) OR (cervical or cervix or cervixes or cervico *) AND (precancer * or cancer * or neoplas * or dysplas * or dyskaryos * or tumor * or tumour * or malignanc * or carcinoma * or adenocarcinoma * or lesion * or squamous or small cell or large cell) OR (cervical intraepithelial neoplasia or CIN or CINII * or CIN2 * or CINIII * or CIN3 * or SIL or HSIL or LSIL or ASCUS or AS-CUS) AND (urin *). We manually searched the relevant publications.

The eligibility criteria included any test-of-accuracy study comparing HPV DNA detection in urine and cervix samples, in women with concern about infection with HPV or development of cervical cancer. If the reference standard was different or not available, we excluded the study. Meta-analysis included studies with data that could be converted into 2 × 2 table. A test’s diagnostic value can be overestimated by certain factors. Therefore, we excluded case-control studies, i.e., studies testing only cervical cancer patients or non-infected patients from the meta-analysis.

### 2.2. Study Extraction, Quality and Selection

For relevant studies, we screened all titles and abstracts. Two reviewers (P.B. and J.S.) independently performed a systematic literature search. In addition, P.B. screened the full texts of the included papers and extracted the subsequent data: characteristics of the study (authors, publication year, country, and purpose), characteristics of the patients (median age and range, cytology and histology results), index test characteristics (volume of sample, storage temperature, DNA extraction and amplification method, test timing as compared to the reference standard). To all studies the quality assessment of diagnostic accuracy studies-2 (QUADAS-2) was applied [26]. The patient selection, index test, standard test, and patient flow were the factors involved in quality evaluation.

### 2.3. Data Synthesis and Statistical Analysis

Upon the detection of any HPV, high-risk HPV, HPV 16 and 18, the 2 × 2 table was made. If the study included more than one method for testing urine HPV, we selected the one with methods closest to those used by other studies. From the estimates, we derived a summary receiver operating characteristic (SROC) curve and the summary accuracy measures with 95% confidence interval (CI) (sensitivity, specificity, likelihood ratio positive and negative (LR+ and LR−)). The shape of a receiver operating characteristic (ROC) curve and the area under the curve (AUC) can help us get a sense of a test’s discriminative power, i.e., AUC presents the measure of diagnostic accuracy. If the curve is located as close as possible to the upper-lefthand corner, and the larger the area under curve, then the test will discriminate better between diseased and healthy individuals. A good indicator of the quality of the test is the area under the curve, which can range from 0 to 1. In a perfect diagnostic test, the AUC is 1, whereas in a nondiscriminating one, the AUC is 0.5 [27]. The forest plots showing the sensitivity and specificity with 95% CI to visualise heterogeneity of studies were generated. In addition, we included the subsequent covariates in meta-regression in order to investigate possible sources of heterogeneity: bias caused by patient selection (high risk versus low risk), purpose (surveillance of HPV versus cervical intraepithelial neoplasia (CIN) and cervical cancer screening), sample timing (urine before versus after cervical tissue collection), storage temperature (more than 0 °C versus less than 0 °C), HPV detection method (conventional PCR versus real time, quantitative polymerase chain reaction (qPCR), DNA microarray, multiplex PCR). 

A meta-analysis of diagnostic test accuracy was conducted using an online, freely available interactive web-based tool: MetaDTA, version 2.01 (https://crsu.shinyapps.io/dta_ma/ (Accessed date: 13 December 2021)). The MetaDTA statistical tool pools the sensitivity and specificity estimates for bivariate random-effects models. This model was fitted as a generalized linear mixed-effect model using the glmer function from the package lme4 of the statistical software R with shiny [28]. This approach accounts for potential threshold effects and covariance between sensitivity and specificity. Using the logit estimates of sensitivity and specificity, the diagnostic odds ratios (DORs) were obtained directly. In addition, using parameters estimated from the bivariate model through the equivalence equations of Harbord et al. [29], the SROC plot was rendered. 

Meta-regression was performed using Meta-DiSc software (version 1.4). To explore sources of heterogeneity in the studies, we used the Moses–Shapiro–Littenberg method by adding covariates to the model [30]. Meta-regression analysis included the threshold effect, weighted least squares method, the inverse of variance of the log of the DOR, and the random effects between studies using restricted maximum likelihood.

Publication bias was conducted using R Studio (version 1.3.959) with “metafor” package. A *p* value < 0.05 was considered statistically significant. 

## 3. Results

**Figure 1:**
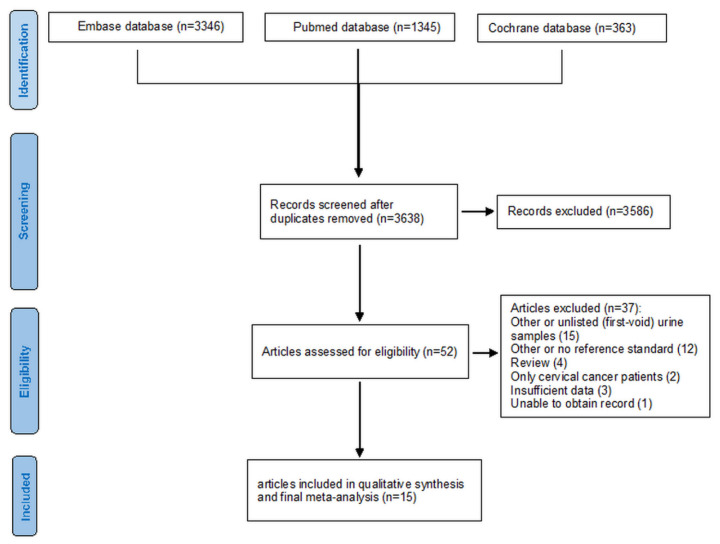
Flow diagram of the studies selected for this meta-analysis.

Identifying and selecting studies is summarized in Figure 1. Of the 5054 potential records, 15 studies (3675 women recruited, 3412 women analysed) were included in the meta-analysis [3,16,17,31,32,33,34,35,36,37,38,39,40,41,42].

### 3.1. Studies Description

The characteristics of included studies in this review and meta-analysis are shown in Table 1 and Table 2. We recruited 8 out of 15 populations of studies from gynaecology or colposcopy clinics, 3 from health centres, 1 from genitourinary medicine and 1 from a general practitioner. In most populations of study, cervical cancer screenings were the purpose of the testing (10/15). Those remaining were for CIN follow-up (3/15) or HPV surveillance (2/15). 

Results of cytological analysis were recorded for 15 populations, i.e., 51% (1706/3360) women had normal conditions, 25% (848/3360) had atypical squamous cells of undetermined significance (ASCUS), 16% (542/3360) had low-grade squamous intraepithelial lesion (LSIL), 0.42% (14/3360) had atypical squamous cells, possible high-grade lesion (ASC-H), and 7.4% (250/3360) had high-grade squamous intraepithelial lesion (HSIL). From the 9 populations with reported histology results, 33.3% (304/912) of women had normal conditions, 25% (229/912) had CIN1, 14.6% (133/912) had CIN2, 1.2% (11/912) had CIN2+, 25% (229/912) had CIN3, and 0.66% (6/912) had histology proved cervical cancer. 

Conventional PCR was used in most studies, but the testing methods used were not uniform. Five of the 15 studies used real-time PCR [31,32,34,40,41], and there was only one PCR-based DNA microarray [37] used out of 15. In one study, real time PCR was evaluated, in the last multiplex PCR. Storage temperatures of urine ranged from −80 °C [33,35,40] to 4 °C [31,32,34,37]. In 13 and 11 studies commercially available amplification platforms and commercial DNA extraction kits, respectively, were used. In all studies, the reference standard of cervical samples for HPV DNA testing were used.

### 3.2. Quality of Studies

**Figure 2:**
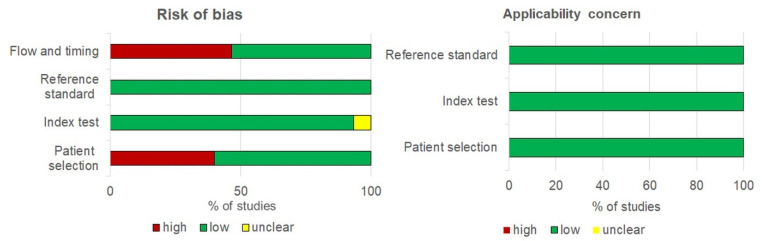
Qualitative assessment of 15 studies included in the meta-analysis using QADAS-2.

A quality evaluation of the studies is shown in Figure 2. Due to narrow patient spectrums for 6 of the studies, the high-risk of bias for patient selection was recorded: 3 studies focused only on patients with CIN of high grade [31,32,39], 2 studies recorded only young women (18–25 age) [16,17], and 1 study included human immunodeficiency virus (HIV) patients [42]. In most studies, the patient flow and timing reduced the risk of bias; 8/15 analysed all recruited participants, and 7 studies analysed (1.9–23.2%) of recruited participants. In 8 of 15 studies, both tests completed during the same day, and in 8 studies, urine samples were collected prior to taking cervical samples. In all low-risk-of-bias studies, the reference standard was applied. Out of 15 studies, 1 used an index test with in-house methods that did not specify a threshold, i.e., the bias of this study was considered unclear risk [33]. In other studies (14/15), a predetermined threshold of the index test with low risk of bias was used. The publication bias did not appear in this study.

### 3.3. Meta-Analysis

**Figure 3:**
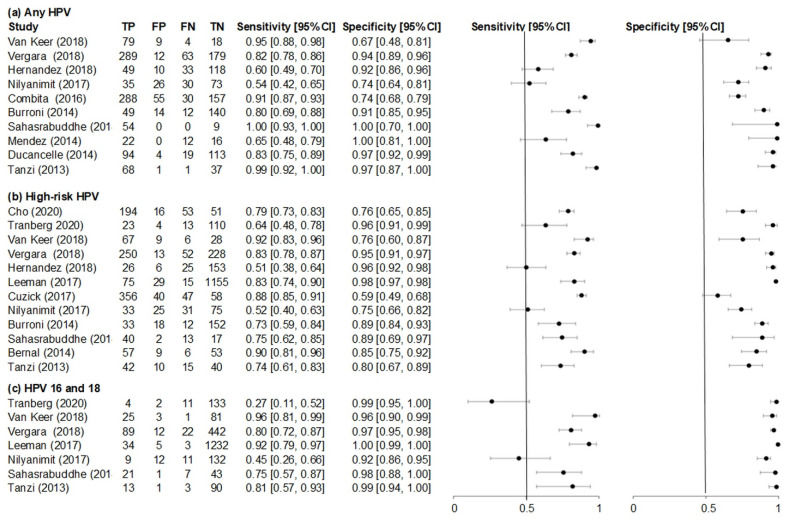
Forest plots of (**a**) any HPV, (**b**) high-risk HPV and (**c**) HPV 16 and 18 sensitivity and specificity for studies evaluating accuracy of first-void urine human papillomavirus (HPV) detection compared to cervical HPV.

The heterogeneity of sensitivity and specificity between individual urine detection studies of any HPV (10 studies), high-risk HPV (12 studies), and HPV 16 and 18 (7 studies) is shown in Figure 3. The individual sensitivities and specificities of any HPV detection in urine varied from 54% [37] to 99% [38,42] and from 67% [29] to 99% [38,39], respectively. Individual sensitivities (51% [30] to 92% [33]) and specificities (59% [36] to 98% [35]) for high-risk HPV detection studies in urine were observed. According to analysis conducted on HPV 16 and 18, sensitivities ranged from 27% [32] to 96% [33] and specificities ranged from 92% [37] to 99% [32,35,42] in urine-detection studies.

**Figure 4:**
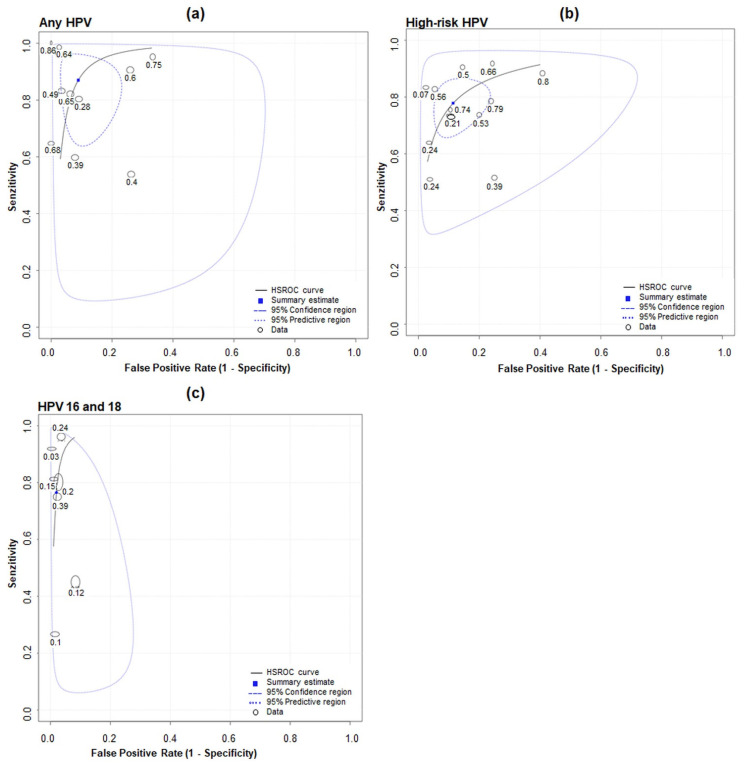
SROC plot for studies evaluating accuracy of detecting (**a**) any HPV, (**b**) high-risk HPV and (**c**) HPV 16 and 18 in first-void urine compared with in cervix.

A SROC plot for pooled sensitivity and specificity for the three groups, (a) any HPV, (b) high-risk HPV and (c) HPV 16 and 18 is shown in Figure 4. Pooled sensitivity for high-risk HPV detection in urine of 78% (70% to 84%) and specificity of 89% (81% to 94%) were calculated. For any HPV detection in urine of 87% (74% to 94%) and 89% (81% to 93%) sensitivity and specificity, respectively, were pooled. HPV 16 and 18 had a pooled sensitivity of 77% (76% to 77%) and specificity of 98% (98% to 98%). The whole upper-left quadrant in Figure 4 represents the 95% prediction region for the SROC plots, i.e., between studies was heterogeneity. For any HPV detection, the 95% prediction region covers the largest portion of the plot, i.e., it had the most heterogeneity between studies (Figure 4a). For any HPV detection, the LR+ was 15.62 (95% CI 4.60 to 53.05) and the LR− was 0.14 (95% CI 0.08 to 0.24). For high-risk HPV detection, the LR+ was 6.81 (4.07 to 11.41) and the LR− was 0.25 (0.18 to 0.34). For HPV 16 and 18 detection, the LR+ was 39.73 (39.33 to 40.14) and the LR− was 0.24 (0.24 to 0.24).

### 3.4. Meta-Regression Analyses

A meta-regression with the following covariates (bias in patient selection, purpose, sample timing, storage temperature and HPV detection method) was conducted to identify the possible sources of heterogeneity. Using the Cochran’s Q test, likelihood ratios and diagnostic odds ratios were tested for homogeneity between studies. Heterogeneity and variation between studies were not confirmed using the covariates listed above (Table 3).

### 3.5. Publication Bias

**Figure 5:**
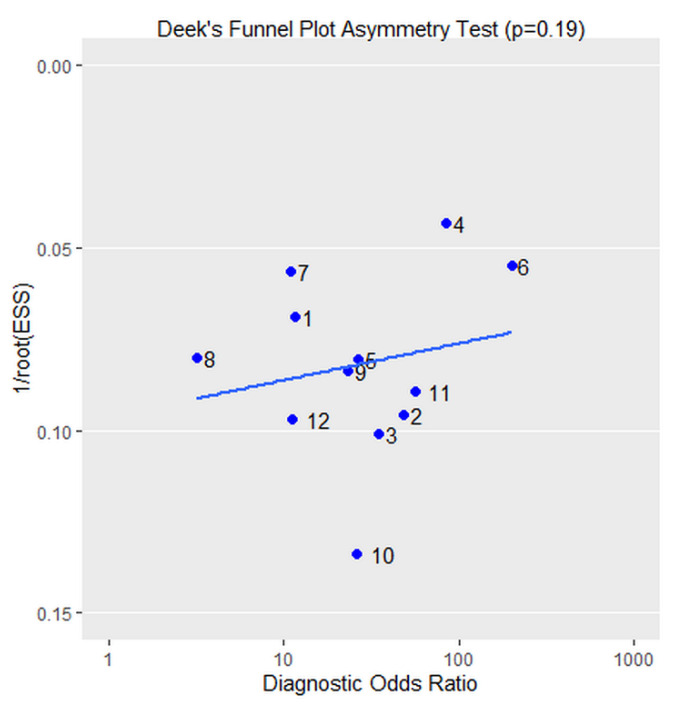
Deek’s funnel plot. The regression test showed no significant publication bias (*p* = 0.19).

We investigated the potential publication bias by using Deek´s funnel plot asymmetry test, as shown in Figure 5. The regression test showed no significant publication bias (*p* = 0.19).

## 4. Discussion

The purpose of diagnostic tests in healthcare settings is to confirm or exclude diagnoses. Assessment of accuracy is determined by comparing the diagnostic test results with the “gold standard” according to which individuals’ true diagnosis can be determined. In our study, the HPV DNA in cervix samples represented the gold standard test, to compare with the HPV DNA in first-void urine samples. 

In Pathak’s review, accuracy of urinary HPV testing for cervical human papillomavirus was investigated through meta-analysis. There was only one source of heterogeneity identified, which was urine sampling, i.e., the accuracy of samples collected as random or midstream, as opposed to first-void samples, decreased by more than 22 times [23]. The first-void urine contains higher levels of high-risk HPV as expected, i.e., 4.8–160 times higher in comparison to the other fraction [24]. The first-void urine can produce more HPV DNA-positive results than paired cervical samples when using sensitive HPV DNA assays [43,44,45]. Therefore, in our meta-analysis we used studies with first-void urine samples.

To evaluate the performance of a diagnostic test, we synthesized sensitivity and specificity from a meta-analysis of diagnostic test accuracy studies. In our meta-analysis, a heterogeneity between the pooled sensitivities and specificities was detected, i.e., pooled sensitivity for high-risk HPV detection in urine was 78% (70% to 84%) and specificity was 89% (81% to 94%). For any HPV detection in urine of 87% (74% to 94%) and 91% (83% to 96%), we pooled sensitivity and specificity, respectively. HPV 16 and 18 had a pooled sensitivity of 77% (76% to 77%) and a specificity of 98% (98% to 98%). 

The bivariate model has been shown to be mathematically identical to the HSROC model when covariates are not included. The HSROC parameters were estimated using parameters of the bivariate model and the equivalence equations of Harbord et al. The SROC plot was drawn using the resulting HSROC parameters [29], and it shows the relationship between sensitivity (y-axis) and 1-specificity (x-axis), illustrating variations in sensitivity and specificity for different thresholds of a test. The whole upper-left quadrant in Figure 4 represents the 95% prediction region for the SROC plots, i.e., between studies there was heterogeneity. For any HPV detection, the 95% prediction region covers the largest portion of the plot, i.e., it had the most heterogeneity between studies (Figure 4a). Regarding the method used in the present meta-analysis, we acknowledge as a limitation that hierarchical models (such as the bivariate model) used in this meta-analysis are likely to be vulnerable when the number of studies is small and also when sample sizes are highly variable, which is partly the case of the present data [46].

The estimates of logit sensitivity and logit specificity were used to calculate LR^+^ and LR^-^. In our study, higher values of the positive likelihood ratio were detected, i.e., for any HPV detection, the LR^+^ was 15.62 (95% CI 4.60 to 53.05) and the LR^-^ was 0.14 (95% CI 0.08 to 0.24). For high-risk HPV detection, the LR^+^ was 6.81 (4.07 to 11.41) and the LR^-^ was 0.25 (0.18 to 0.34). For HPV 16 and 18 detection, the LR^+^ was 39.73 (39.33 to 40.14) and the LR^-^ was 0.24 (0.24 to 0.24).

QUADAS-2 was used as a revised tool for the quality assessment of diagnostic accuracy studies [26]. The patient selection, index test, standard test, and patient flow were the factors involved in quality evaluation. Generally, these studies had a high quality, i.e., an appropriate patient spectrum and a consecutive or random recruitment of participants were used, the majority of recruited participants were included in analyses and all of them used the same reference standard. However, the main weakness in some studies was that they included only patients with CIN2+ [31,32,39], young women (18–25) [16,17] and HIV patients [42]. In addition to resulting in a high prevalence, these factors could also lead to a biased evaluation of test accuracy [47,48].

To determine whether these differences in testing methods influenced results, a meta-regression was used. In the meta-regression analysis, the variation in accuracy was not seen by using covariates (bias in patient selection, purpose, sample timing, storage temperature, and HPV detection method). However, a heterogeneity between the pooled sensitivities and specificities, and higher values of the positive likelihood ratio were detected. These factors could have a significant impact on the probability of infection in HPV-positive women. Therefore, the false positive results could lead to unnecessary invasive examination and costs, which is the advantage of the urine-testing method. However, the high specificity of our test suggests that this scenario is less likely to occur. For these reasons, our results should be interpreted cautiously because there is always the risk of over- or underestimating data. Testing methods need to be more consistent and reproducible if the test is to be successfully implemented in current practice. Therefore, we recommend standardizing urine testing methods, i.e., before incorporating urine testing for HPV into cervical cancer screening guidelines, it is important to minimise variation.

Based on the above-mentioned facts, it is necessary to optimise the HPV DNA detection in first-void urine in order to minimise variation of the first-void urine test (sensitivity and specificity) for the presence cervical HPV in women. Optimised HPV DNA detection in urine should include the following: (1) use of the first-void urine (morning or later during the day) captured with a urine collection device [49]; (2) immediately mix first-void urine with a conservation medium to prevent HPV DNA degradation during extraction and storage; (3) provide sufficient first-void urine volume for subsequent sample concentration; (4) recover cell-associated HPV DNA as well as cell-free DNA [43]; (5) use of HPV tests meeting the criteria for primary cervical cancer screening [50]; (6) not cleaning the genital area before collecting the sample [21]; and (7) collect the first-void urine samples before cervical samples since this may reduce mucus and debris [51].

## 5. Conclusions

Our meta-analysis demonstrates the accuracy of detection of HPV in urine for the presence of cervical HPV. Although progress is continuously made in urinary HPV detection, further studies are needed to evaluate and to improve the accuracy of the first-void urine test in order to be comparable with other screening methods. Different testing platforms and conditions were used in these studies. Therefore, all results should be interpreted carefully, as they may have been over- or underestimated.

## Figures and Tables

**Table 1:** Qualitative characteristics of included studies.

Author, Year, [Ref]	Country	Study Context (Purpose)	Cytology (Histology)	Timing	HPV Detection Method	DNA Extraction Method	DNA Amplification Platform	Primers
Hyun-Woong Cho, 2020, [31]	South Korea	colposcopy (follow-up of CIN)	abnormal (CIN2, CIN3, cervical cancer)	urine after cervical	real-time PCR	QIAamp DNA blood minikit	Seegene	L1
Mette Tranberg, 2020, [32]	Denmark	general practitioner (cancer screening)	ASC-US (normal, CIN1, CIN2+)	another day, urine after cervical	real-time PCR	MagNA Pure LC total nucleic acid isolation kit	Roche	L1
Severien Van Keer, 2018, [33]	Belgium	colposcopy (HPV surveillance)	NILM, ASCUS/LSIL, ASC-H/HSIL (normal, CIN1, CIN2, CIN3)	same day, urine before cervical	qPCR	Non-commercial	—	—
Nicolás Vergara, 2018, [3]	Chile	primary health care centre (cancer screening)	normal, ASC-US, HSIL, LSIL (—)	same day, urine before cervical	conventional PCR	—	Agilent Technologies	L1/PGMY 09/11
Brenda Y. Hernandez, 2018, [34]	Yap	community health centre (cancer screening)	normal, ASC-US, HSIL, LSIL (normal, CIN2, CIN3, cervical cancer)	urine before cervical and urine after cervical	real-time PCR	—	Roche (Linear Array)	L1/PGMY 09/11
A Leeman, 2017, [35]	Spain	colposcopy (follow-up of CIN)	normal, ASCUS/LSIL, ASC-H/HSIL (normal, CIN1, CIN2, CIN3)	same day, urine before cervical	conventional PCR	—	Innogeneticstechnology	L1/SPF10
Jack Cuzick, 2017, [36]	United Kingdom	colposcopy (follow-up of CIN)	ASCUS, LSIL, HSIL (normal, CIN1, CIN2, CIN3, cervical cancer)	same day, urine before cervical	conventional PCR	QIAamp DNA Mini Kit	—	E1
Pornjarim Nilyanimit, 2017, [37]	Thailand	(cancer screening)	normal, LSIL, HSIL (—)	urine after cervical	PCR based DNA microarray	HPV GenoArray Diagnostic Kit	HybriBio	L1
Alba Lucía Combita, 2016, [17]	Colombia	health center (cancer screening)	normal, ASCUS/LSIL, ASC-H/HSIL (—)	same day, urine before cervical	multiplex PCR	NucliSENS easyMAG Extraction Kit	Luminex technology	E7
Elena Burroni, 2014, [16]	Italy	(cancer screening)	normal, ASCUS/LSIL, ASC-H/HSIL (—)	8 days (median), urine after cervical	conventional PCR	QIAamp DNA Mini Kit	Innogenetics	L1
Vikrant V. Sahasrabuddhe, 2014, [38]	USA	colposcopy (cancer screening)	NILM, ASCUS/LSIL, HSIL (normal, CIN1, CIN2, CIN3)	same day, urine before cervical	conventional PCR	QIAamp DNA Blood Kit	Roche (Linear Array)	—
Keimari Mendez, 2014, [39]	USA	gynaecology clinic (cancer screening)	ASCUS/LSIL, ASC-H/HSIL (CIN1, CIN2)	same day, urine before cervical	conventional PCR	MagNA PureDNA Isolation Kit 1	Roche (Linear Array)	—
A. Ducancelle, 2014, [40]	France	colposcopy (cancer screening)	normal, ASCUS/LSIL, HSIL (-)	—	real-time PCR	QIAamp viral RNA mini kit	Innogenetics	L1
Samuel Bernal, 2014, [41]	Spain	gynaecology clinic (HPV surveillance)	normal, ASCUS/LSIL, HSIL (normal, CIN1, CIN2, CIN3)	same day, urine before cervical	real-time PCR	Cobas X 480	Roche	—
Elisabetta Tanzi, 2013, [42]	Italy	genitourinary clinic (cancer screening)	normal, ASCUS/LSIL, HSIL (-)	same day	conventional PCR	BioMérieux NucliSENS1 MiniMAG1	Innogenetics	L1(MY09/MY11)

**Table 2:** Quantitative characteristics of included studies.

Author, Year [Ref]	Sample Recruited (Sample Detection)	Median Age (Range)	Normal	ASCUS/LSIL	ASC-H/HSIL	Normal	CIN1	CIN2 (CIN2/3)	CIN3 (Cancer)	First-Void Urine (Volume Analysed in mL)	Storage Temperature in °C
Hyun-Woong Cho, 2020, [31]	314 (314)	40 (20–60)	─	244/─	─/70	─	─	21	104 (4)	(30)	4
Mette Tranberg, 2020, [32]	150 (150)	45 (30–59)	─	150/─	─	11	10	11		(10–12)	4
Severien Van Keer, 2018, [33]	110 (110)	36 (25–64)	58	36/─	─/15	7	11	6	9	(median; 19)	−80
Nicolás Vergara, 2018, [3]	543 (543)	(18–64)	483	24/22	─/12	─	─	─	─	(10–15)	−20
Brenda Y. Hernandez, 2018, [34]	217 (210)	(21–65)	179	31/3	─/4	2	─	2	5 (2)	(30)	4
A Leeman, 2017, [35]	113 (91)	(18–60)	28	11/28	9/15	50	22	13	6	(16)	−80
Jack Cuzick, 2017, [36]	652 (501)	30 (18–69)	─	160/292	─/49	185	99	64	79	(0.5)	─
Pornjarim Nilyanimit, 2017, [37]	164 (164)	(19–69)	95	─/50	─/19	─	─	─	─	(15)	4
Alba Lucía Combita, 2016, [17]	540 (530)	(18–25)	462	45/17	2/1	─	─	─	─	(9)	−20
Elena Burroni, 2014, [16]	271 (215)	25	205	3/4	1//1	─	─	─	─	(60)	−20
Vikrant V. Sahasrabuddhe, 2014, [38]	72 (72)	28 (20–61)	18	23/11	─/16	17	28	16	10	(0.53)	20
Keimari Mendez, 2014, [39]	52 (50)	(21–60)	─	27/13	2/5	─	42	7	─	(6)	−20
A. Ducancelle, 2014, [40]	245 (230)	(18–55)	34	70/59	─/25	─	─	─	─	(1)	−80
Samuel Bernal, 2014, [41]	125 (125)	36 (21–65)	65	21/22	─/14	43	17	4	16	(20)	─
Elisabetta Tanzi, 2013, [42]	107 (107)	42 (22–70)	79	3/21	─/4	─	─	─	─	(15)	−20

**Table 3:** Multivariate meta-regression results for characteristics with backward regression analysis.

Meta-Regression (Inverse Variance Weights ^1^)					
Var.	Coeff.	Std. Err.	*p*-Value	RDOR ^2^	(95% CI)
Cte.^3^	4.202	0.900	0.006		
S ^4^	−0.628	0.307	0.096		
Bias in patient selection	0.170	0.866	0.852	1.19	(0.13; 10.98)
Purposes	−2.708	1.759	0.184	0.07	(0.00; 6.13)
Sample timing	−0.318	1.056	0.776	0.73	(0.05; 11.00)
Storage temperature	0.269	1.068	0.811	1.31	(0.08; 20.39)
HPV detection method	1.556	1.451	0.333	4.74	(0.11; 197.51)

^1^ Variables were retained in the regression model if *p* < 0.05. ^2^ Relative diagnostic odds ratio. ^3^ Constant coefficient. ^4^ Statistic S.

## Data Availability

The data presented in this study are available on request from the corresponding author.
